# Broad-Spectrum Antibacterial Effects of Human Adipose-Derived Stromal Cells

**DOI:** 10.1155/2019/5389629

**Published:** 2019-10-29

**Authors:** Paul Monsarrat, Philippe Kémoun, Louis Casteilla, Valérie Planat-Bénard

**Affiliations:** ^1^The Department of Oral Rehabilitation, Paul Sabatier University, Faculty of Dentistry, Toulouse University Hospital (CHU de Toulouse), Toulouse, France; ^2^STROMALab, Université de Toulouse, CNRS ERL 5311, EFS, ENVT, Inserm U1031, UPS, Toulouse, France; ^3^The Department of Oral Surgery, Periodontology and Oral Biology, Paul Sabatier University, Faculty of Dentistry, Toulouse University Hospital (CHU de Toulouse), Toulouse, France

## Abstract

**Introduction:**

Many pathological conditions may benefit from cell therapy using mesenchymal stromal cells, particularly from adipose tissue (ASCs). Cells may be grafted in an environment with a remnant polymicrobial component. The aim is to investigate the behavior of ASCs when brought in contact with a large panel of bacteria.

**Materials and Methods:**

Carboxyfluorescein-labelled bacterial interaction with ASCs was followed by confocal time-lapse microscopy. Costaining with LAMP-1 was also analyzed. Viability of 4 gram-negative and 4 gram-positive bacterial strains after 6 h of coculture with ASCs was assessed by agar colony counting and by flow cytometry using SYTO-62®/propidium iodide (PI) for membrane permeabilization and DiOC6 for depolarization. A murine model of periodontitis was used to assess *in vivo* antibacterial capacities of ASCs.

**Results:**

A significant increase of PI-positive events for all bacterial strains and an increase of the DiOC6 signal were obtained after contact with ASCs. The number of CFU was also significantly decreased for several bacterial strains. 0.4 *μ*m transwell systems illustrated the necessary direct contact to induce maximal bacterial membrane damages. Some bacteria were observed into phagolysosomes, confirming macrophage-like properties of ASCs. *In vivo*, the bacterial load was significantly lower in the ASC-grafted side compared to the control.

**Conclusion:**

Our results highlight for the first time a broad range of antibacterial actions of ASCs, by phagocytosis, secretion of oxygenated free radicals and antibacterial molecules. These data are in line with the development of new therapeutic strategies based on ASC transplantation, appropriated in immune-dysbiotic tissue context such as periodontitis or chronic wounds.

## 1. Introduction

Bacterial infections are a major public health issue of our society with a significant impact in terms of direct and indirect costs, and the increased resistance of bacteria to antibiotics also requires reflection on new therapeutic strategies [1]. Human polymicrobial infections are involved in burn or diabetic foot wound, in chronic lung infection, or in periodontal diseases [2]. It is also estimated that two-third of infections involved biofilm formation, constituted of bacterial communities embedded into a matrix of extracellular polymeric saccharides, leading to particular resistance to antibiotics [1].

Oral pathologies, in particular, periodontitis, are considered as the archetype of bacterial diseases involving biofilms, and several hundred bacterial species could be identified into an oral ecosystem [1]. Periodontitis is a dysbiotic chronic inflammatory disease of the soft and hard tissues supporting the teeth, affecting 15-50% of adults in developed countries [3]. This is even more important than periodontitis affecting general health and overall quality of life [3]. If untreated, this could lead to the formation of deep infrabony defects and soft tissue crevices called “periodontal pockets” between the tooth and its bony socket [4]. Bacterial ecology undergoes complex spatial and temporal changes [5]; the establishment of primary colonizers (e.g., *Streptococcus* spp.), offering attachment sites to other bacteria, allows a shift of a gram-positive aerobic into a gram-negative anaerobic flora (e.g., *Fusobacterium nucleatum*, *Porphyromonas gingivalis*). In this context, the regeneration of a bone, cement, and functional periodontal ligament remains a challenge. The objective of complete periodontal apparatus regeneration is rarely achieved, and current periodontal therapies give poor predictability [4]. Current therapies fail to address simultaneously the need for *ad integrum* regeneration of all periodontal tissues, the persistence of a low-noise residual inflammatory context, and a residual bacterial component inherent in any treatment of the tooth-supporting tissues [4]. Moreover, some genetic features, physiological parameters (*e.g.*, age, sex), local contexts (*e.g.*, infection, scars), and systemic diseases may also compromise the regenerative potential [6]. It seems obvious to develop new strategies to reverse dysbiosis at the same time as promoting tissue regeneration.

Mesenchymal stromal cells (MSCs) as effector of a cell therapy approach provide alternative options as demonstrated by the wide current diversification of the fields of applications [7]. Among their broad range of action, antibacterial capacities of MSCs against some pathogens have recently been revealed [8]. Adipose-derived mesenchymal stromal cells (ASCs) are particularly good candidates for tissue engineering. The angiogenic properties of ASCs gave promising results in skin ulcers with severe vascular disease, in part by their differentiation into endothelial-like cells, a secretion of proangiogenic factors [9, 10], and their immunomodulation capacities [7]. Furthermore, the use of ASCs in the context of periodontal regeneration is currently investigated in animal models [4]. Thus, the provision of ASCs within an environment that remains a bacterial component such as periodontitis or chronic wounds requires studying their potential behavior. Transcriptional analyses reveal that ASCs share the expression of a great number of genes with macrophages, in particular, related to endocytosis, actin remodeling, and vesicle trafficking [11]. Moreover, ASCs are able to internalize yeast *Candida albicans* and to exhibit some microbicide activities [12].

Although the antibacterial effect has been reported on a limited number of bacterial strains from bone marrow-derived MSCs, very few data are available for ASCs. Thus, a better understanding of the interaction of ASCs with bacteria is required to better anticipate the benefits of these cells in dysbiotic environments. In this study, we therefore adopted an original and comprehensive approach to investigate the behavior of ASCs when they were brought in contact with gram-negative and gram-positive bacteria. The ASC antibacterial effect was studied on 8 bacterial strains, representative of a wide range of gram-negative and gram-positive bacteria, some of which are pathogens found in chronic wounds or periodontitis. We also considered several mechanisms and explored both a possible direct and indirect antibacterial action.

## 2. Materials and Methods

### 2.1. Bacterial Culturing and Preparation

[Supplementary-material supplementary-material-1] presented all tested strains, either from ATCC or from CIP collection. The three periopathogens (*Fusobacterium nucleatum* (*Fn*), *Porphyromonas gingivalis* (*Pg*), and *Prevotella intermedia* (*Pi*)) were cultured onto trypticase soy agar plates supplemented with 10% sheep blood, hemin (5 *μ*g/mL), and menadione (1 *μ*g/mL) and maintained into anaerobic atmosphere (GENbox anaer, Biomérieux, Marcy l'Etoile, France). *Streptococcus sanguinis* (*Sg*), cultivated onto blood agar, was maintained into aerobic atmosphere. *Enterococcus faecalis* (*Ef*) was anaerobically kept onto brain heart infusion agar plates (BHI). Indeed, *Lactobacillus casei* (*Lc*), *Staphylococcus aureus* (*Sa*), and *Escherichia coli* (*Ec*) were cultured onto BHI agar aerobically. Incubation was performed at 37°C.

For cell infection, bacteria were cultured overnight in a Wilkins-Chalgren broth. Bacteria were centrifuged 10 minutes at 1750 g, then washed into phosphate saline buffer (PBS). Optical density at 600 nm (OD600) was measured, and strains were appropriately diluted in culture medium *α*-minimum essential medium (*α*-MEM, Life Technologies) containing 10% decomplemented fetal calf serum (FCS). The initial dose of bacteria was previously calculated to obtain a 6 to 7 × 10^6^ colony-forming units (CFUs) at the end of experiments (after 6 hours of incubation at 37°C in cell culture medium).

For wild flora, human subgingival dental plaque was sampled using a paper point inserted behind the gingiva of patients with periodontitis for 10 seconds. Samples were transported at room temperature to the laboratory in the semisolid Amies Transport Medium with charcoal and processed. After serial dilution, bacteria were cultured anaerobically for 6 hours, with and without ASCs. Anaerobic bacterial colonies were then counted, as detailed below.

### 2.2. ASC Culturing

Inguinal subcutaneous adipose tissue samples were obtained from donors undergoing elective abdominal dermolipectomy with no objection certificate according to the bioethic law no. 2004-800 of August 6, 2004, and were processed as previously described to isolate ASCs [10]. Briefly, adipose tissues were digested at 37°C for 45 minutes in phosphate-buffered saline (PBS) containing 2% fetal calf serum (FCS) and 0.8 U/mL collagenase NB4 (Serva, Heidelberg, Germany), filtrated at 25 *μ*m, and then centrifuged at 600 g for 8 minutes, to remove mature adipocytes. Red blood cells were lysed into buffer containing 140 mM NH_4_Cl and 20 mM Tris for 5 minutes at 4°C. Cells were centrifuged at 600g for 5 minutes, and this vascular stromal fraction was seeded at 4000 cells/cm^2^ in *α*-MEM with 10% FCS, 0.25 *μ*g/mL amphotericin, 100 *μ*g/mL streptomycin, and 100 UI/mL penicillin and maintained in 5% CO_2_ atmosphere. To be sure to have no effect of a possible gradual release of these antibiotics or antifungals [13], cells were washed the next day with PBS, maintained, and subcultured in culture medium without antifungal and antibacterial products. Similarly, to prevent from a direct antibacterial effect of the serum complement system, the fetal calf serum used was decomplemented. The medium was renewed every 2 to 3 days. Cells were used from passages 1 to 3. Characteristics of donors used in this study were described in [Supplementary-material supplementary-material-1].

### 2.3. Assessment of Bacterial Recovery on Agar

To enumerate bacterial colonies, we used a 6 × 6 drop plate procedure. Samples were serially diluted using a multichannel pipette in a 96-well plate, and 10 *μ*L drops were deposited by inverted pipetting onto appropriate agar. Plates were allowed to dry, then placed into an incubator. After adequate incubation time, bacterial CFUs were counted, taking into account the dilution factor, and expressed as logarithm base 10.

### 2.4. Assessment of Bacterial Membrane Properties by Flow Cytometry

After incubation time, supernatants were flushed several times and 50 *μ*L was collected for staining in 200 *μ*L PBS containing propidium iodide (Sigma-Aldrich) and Syto-62© (Life Technologies) at final concentration of, respectively, 20 *μ*M and 1 *μ*M. For assessing membrane potential, supernatants were stained with 3,3′-dihexyloxacarbocyanine iodide (DiOC6(3), 1 *μ*m, Sigma-Aldrich) [14] and Syto-62©. All experiments were analyzed by flow cytometry using FACSCalibur (BD Biosciences, Le Pont de Claix, France).

### 2.5. Scanning Electron Microscopy

Cells were cultured onto sterile plastic 18 mm × 18 mm coverslips until they reached 60-70% confluence and incubated with bacteria for 6 hours. Cells or bacteria were fixed in Sorenson's buffer containing 2% glutaraldehyde for at least 4 h at 4°C. After 12-hour wash in 0.1 M sodium cacodylate buffer, samples were dehydrated in a graded ethanol series, dried by critical point drying with EMSCOPE CPD 750, and coated with a thin layer of platinium of 2 nm in a sputter coater (Leica, Nanterre, France). Samples were then observed with the ESEM Quanta 250 FEG (FEI, Hillsboro, Oregon, USA) at an accelerating voltage of 5 kV.

### 2.6. Transmission Electron Microscopy

Fixed samples, as described above, were postfixed with 1% OsO_4_ in Sorensen's buffer for 1 hour followed by dehydration in ethanol and propylene oxide, then embedded in epoxy resin (Epon 812). Ultrathin sections (70 nm) were mounted on 100 mesh collodion-coated copper grids and counterstained with 3% uranyl acetate in 50% ethanol and with 8.5% lead citrate before being examined on an HT 7700 Hitachi at an accelerating voltage of 80 kV.

### 2.7. Assessment of Necessary Contact between Bacteria and ASCs

After 6 hours of incubation, culture medium (with or without cells, with or without bacteria) was collected and filtered at 0.22 *μ*m and frozen at -20°C to eliminate residual bacteria. Aliquots of 90 *μ*L of medium were transferred to a 96-well plate and infected with 10 *μ*L of culture medium to reach the required concentration of bacteria. Plates with 12 wells and 0.4 *μ*m PET membrane inserts (Merck Millipore, Darmstadt, Germany) were used for transwell assays. Either the inner or the outer part of the transwell assay was infected, with or without ASCs ([Supplementary-material supplementary-material-1]). We compared bacteria from the inner part with cells versus without cells (indirect contact) and bacteria from the outer part with cells versus without cells (direct contact). After 6 hours of incubation, membrane permeability was assessed by flow cytometry as described above.

### 2.8. ROS Measurements

Cells at 80% confluence were stained with 4 *μ*m diacetoxymethyl ester 6-carboxy-2′,7′-dichlorodihydrofluorescein diacetate (H2DCFDA, Life Technologies) for 30 minutes in PBS at 37°C. Cells were then recovered in culture medium for 30 minutes before being exposed to bacterial solution or the control. Bacteria were added at a 1 : 100 MOI for 45 minutes or at different time points. For inhibitors, they were added 15 minutes after cell recovering has begun for a total of 45 minutes, then during totality of incubation time. Cells were trypsinized, and green fluorescence was immediately recorded by flow cytometry. The following inhibitors were tested: the antioxidant N-acetyl cysteine (4 mM, Sigma-Aldrich, Lyon, France), the SOD mimetic and peroxynitrite scavenger with catalase-like activity MnTBAP (50 *μ*M, Calbiochem, Merck Millipore), and the actin polymerization inhibitor cytochalasin D (0.4 *μ*M, Sigma-Aldrich).

### 2.9. Confocal Visualization

Before use, bacteria were stained by carboxyfluorescein diacetate succinimidyl ester (CFDA-SE, Sigma-Aldrich). After centrifugation, bacteria were incubated for 1 hour in PBS containing 20 *μ*M CFDA-SE at RT in the dark. After washing twice in PBS, bacteria were incubated for further 30 minutes to allow the efflux of the staining solution not covalently fixed, then washed twice. Counting was performed by flow cytometry using microbeads (AbC® Anti-Mouse Bead Kit, Invitrogen) as reference. For phagocytosis assessment, 8-well PCA slide chambers (Sarstedt, Marnay, France) were coated with 0.1% gelatin solution. Cells were seeded and cultured as indicated to reach 70% confluence. Infection by *Fn* and *Sg* was performed at a 1 : 100 multiplicity of infection (MOI) for 1 hour. Cells were fixed in 3.7% paraformaldehyde for 15 minutes, then permeabilized by 0.3% Triton X100 for 20 minutes. Slides were blocked with 1% bovine serum albumin (BSA) solution in PBS for 30 minutes at room temperature (RT). Primary antibodies mouse anti-LAMP-1 (H4A3, DSHB) were incubated at 1 : 100, 1-hour RT and washed 3 times for 5 minutes with PBS. In negative controls, primary antibodies were replaced by mouse IgG1 isotype antibody. Secondary antibodies anti-mouse DyLight 650 (1 : 200) were incubated 1-hour RT and washed 3 times for 5 minutes with PBS. Cell nuclei were stained by Hoechst 33342 (5 mg/mL) for 30 minutes, washed, and mounted with Dako fluorescence mounting medium (Dako, Glostrup, Denmark). Fluorescence staining was visualized by confocal microscopy (ApoTome, Zeiss). For time-lapse analyses, 8-well PCA slide chambers were seeded with ASCs cultivated until 70% confluence. Cells were previously stained with CellTrace® Far Red (Life Technologies, Saint-Aubin, France) at 2 *μ*M then infected by CFDA-SE conjugated *Sg* at a 1 : 100 MOI. Cells were monitored for 14 hours by spinning disk (Nikon, Champigny-sur-Marne, France).

### 2.10. Phagocytosis Assay

Bacteria were previously labelled with 1 mM pHrodo® Green STP Ester (Molecular Probes, Life Technologies) according to the manufacturer's recommendations (including methanol washing steps). As the negative control (isotype), exactly the same procedure was followed for noninoculated WC broth. Cells were cultivated until 80% confluence onto 6-well plates and then infected at 1 : 100 MOI for 1 hour. To demonstrate phagocytosis, comparison was performed versus cells treated with 0.4 *μ*M cytochalasin D for 45 minutes before and during the time of infection. Cells were then trypsinized, recovered in PBS with 2% BSA, and the percentage of positive cells for green fluorescence was immediately measured by flow cytometry.

### 2.11. Periodontitis Mouse Model

An original model of periodontal lesion in mice induced by oral gavage of periodontal pathogenic bacteria was used as previously described [4, 15]. A mixture of periodontopathogenic bacteria (*Porphyromonas gingivalis*, *Fusobacterium nucleatum*, and *Prevotella intermedia*) was brought repeatedly for 1 month to the molar regions to induce periodontal lesions. The ASCs were brought in a collagen solution on one molar side or only the collagen solution in the contralateral molar side. Six mice were sacrificed at time 0 then at 1 and 6 weeks.

### 2.12. Statistical Analyses

Results were expressed as the mean ± SEM of at least five human donors of ASCs during at least three experiments. Comparisons between conditions with and without ASCs were performed by the nonparametric Wilcoxon test. For multiple comparisons (multiple time points or doses), statistics were corrected by multiple comparisons using Bonferroni adjustment. Correlation was analyzed by Spearman's test. The level of significance was set to .05. Multivariate analysis was performed using multilevel mixed-effects linear regression. Graphics and statistics were performed using Stata 13.1 (StataCorp, College Station, TX, http://www.stata.com). For bacterial growth modeling, the Gompertz model was used via the R software to estimate the parameters.

## 3. Results

### 3.1. ASCs Exhibited a Rapid Antibacterial Effect

For the four strains tested (*Lc*, *Ec*, *Sg*, and *Sa*), we consistently observed a significant decrease in the number of CFUs when bacteria were brought into contact with ASCs. This effect was found to be maximal at 6 hours, whereas a trend can be seen at 4 and 9 hours ([Supplementary-material supplementary-material-1]). Proportion of propidium iodide- (PI-) positive bacteria also suggested a maximum of permeabilized bacteria at 24,000 to 48,000 cells by well in 12-well plates ([Supplementary-material supplementary-material-1]), allowing us to define the working condition at 80% confluence and 6-hour incubation.

### 3.2. Broad-Spectrum Antibacterial Effect of ASCs Was Dependent on the Initial Bacterial Load

**Figure 1:**
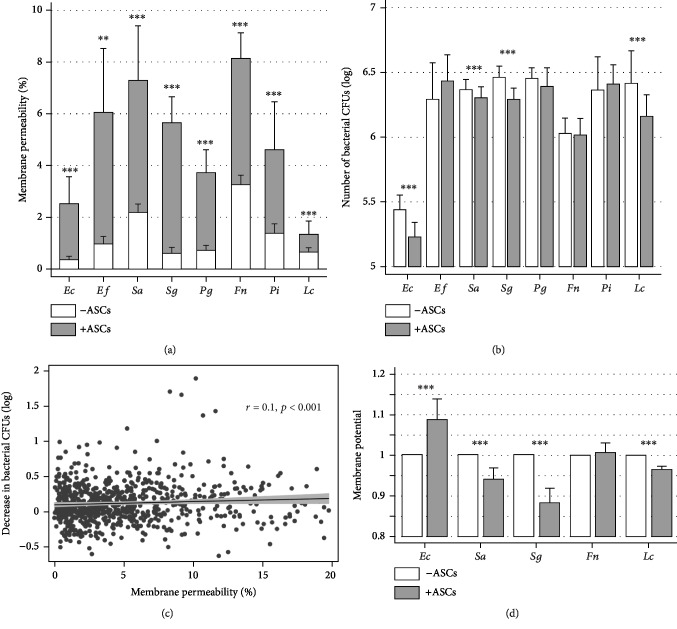
ASCs exhibited broad-spectrum antibacterial activity for both gram-positive and gram-negative strains. Several bacterial strains were brought in contact for 6 hours without (blank bars) or with (black bars) ASCs aerobically or anaerobically. (a) Bacteria were stained Syto-62® and propidium iodide (PI), then analyzed by flow cytometry. Changes in the proportion of PI-positive bacteria reflected changes in bacterial wall permeability (*N* = 10). (b) After serial dilutions, bacteria were incubated onto agar for CFU counting with or without ASCs (*N* = 10). (c) Scatter graph represented changes in CFU number versus proportion of PI-positive bacteria. A linear regression was represented with the corresponding correlation coefficient. (d) After labelling with DiOC6(3), the green/red ratio was used to analyze modification of bacterial membrane polarization independently from the change of bacterial size and shape (*N* = 12). ^∗^*p* < .05, ^∗∗^*p* < .01, and ^∗∗∗^*p* < .001 between conditions with and without ASCs.

For all strains, the proportion of PI-positive bacteria was significantly increased after contact with ASCs (Figure 1(a) and [Supplementary-material supplementary-material-1]). The number of CFUs for *Ec*, *Sa*, *Sg*, and *Lc* was also significantly decreased after 6 hours of contact with ASCs (Figure 1(b)). When plotted, we observed a significant correlation (*r* = 0.1, *p* < .001) between the decrease of CFUs and proportion of PI-positive bacteria (Figure 1(c)). Thus, most experiments were assessed using PI-positive bacteria as an outcome. [Supplementary-material supplementary-material-1] demonstrated that the initial dose of bacteria influenced the capacity of ASCs to induce antibacterial action, revealing a more bactericide than bacteriostatic action. The decrease in the red/green ratio for DiOC6(3) staining of bacteria (independently of their size and shape change ([Supplementary-material supplementary-material-1], [Supplementary-material supplementary-material-1])) revealed that ASCs could be able to induce a significant modification of bacterial membrane polarization (Figure 1(d)). Considering ASC characteristics, a multivariate analysis showed that the percentage of PI-positive events of *Fn* were significantly increased when the body mass index increased, regardless of age, number of bacteria, and its percentage of PI-positive events in the control group (coefficient of 2.18 with a 95% confidence interval of [0.78; 3.57] and a number of 135 observations for 37 patients).

### 3.3. ASCs Could Disturb Bacterial Division

**Figure 2:**
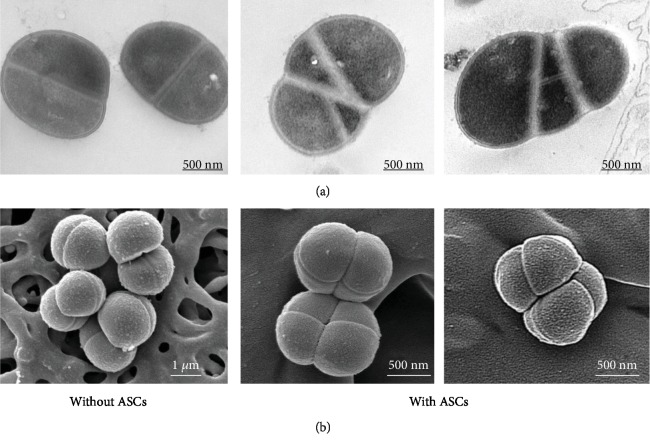
ASCs modified bacterial growth kinetics by targeting the membrane. (a) Transmission electron microscopy highlighted multiple bacterial division planes when bacteria *Sa* were brought into contact with ASCs. No difference in membrane thickness was detected (without ASC: 45.7 nm ± 7.2 and with ASC: 51.5 nm ± 13.6). (b) Scanning electron microscopy confirmed pseudo multicellular formations of *Sa* when incubated with ASCs.

Taking microbeads as FSC/SSC reference, we observed significant changes in size and granularity of bacteria after contact with ASCs. For example, FSC and SSC were increased for *Sg* and *Fn* and decreased for *Sa* ([Supplementary-material supplementary-material-1]). After 6-hour contact with cells, we measured kinetic growth of bacteria by optical density change using the modified Gompertz equation. For three strains *Ec*, *Sa*, and *Sg*, we recorded a significant decrease in the growth rate (*μ* parameter) after contact with ASCs whereas other parameters were not significantly modified (Table 1). We observed no apparent morphological changes of bacteria (Figures 2(a) and 2(b), [Supplementary-material supplementary-material-1]), both in SEM or TEM (such as visible holes or blebs). We confirmed that there was no bacterial wall disruption on *Ec* to allow the passage of *β*-galactosidase ([Supplementary-material supplementary-material-1]). Bacterial wall thickness of *Sa* was not significantly modified (Figure 2). Nevertheless, we encountered abnormalities of *Sa* division after ASC contact, with the presence of several focal planes of division by bacteria, leading to pseudo multicellular formations (Figures 2(a) and 2(b)).

### 3.4. A Direct Contact with ASCs Was Necessary to Induce Bacterial Permeabilization

**Figure 3:**
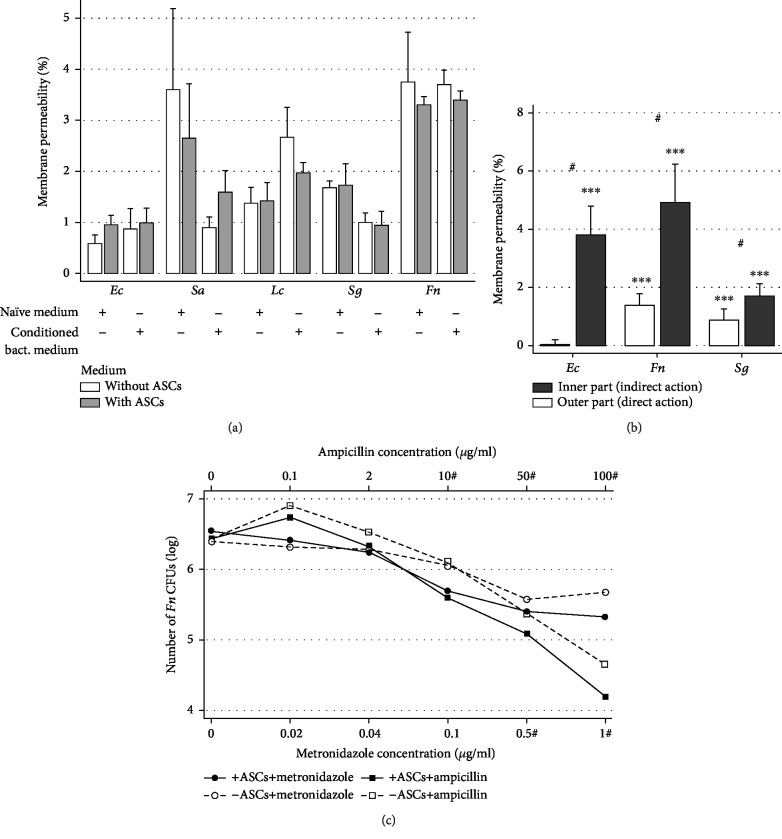
A direct contact with ASCs was needed to induce bacterial permeabilization, which can be used to potentiate the antibiotic effects. (a) Medium without ASCs (white bars) or with ASCs (grey bars) was obtained after 6 hours of incubation without bacteria (naïve medium) or with bacteria (bacterial conditioned medium). After adequate preparation, the culture medium was reinfected with bacteria to the required concentration again for 6 hours (*N* = 5). (b) Either the inner (white) or the outer (black) part of 12 0.4 *μ*m well inserts were infected with 3 strains (*Ec*, *Fn*, and *Sg*). ^∗^*p* < .05, ^∗∗^*p* < .01, and ^∗∗∗^*p* < .001 significantly increased membrane permeability; ^#^*p* < .05 between the internal and external parts (*N* = 7). (c) The *Fn* strain, chosen because there was no inhibitory effect of ASCs on agar, was exposed to different concentrations of two antibiotics (ampicillin or metronidazole), with or without ASCs for 6 hours. ^#^*p* < .05 shows a significant difference between -ASCs and +ASCs for a given time, after adjustment for multiple comparisons (*N* = 7).

After 6 hours of bacterial incubation with ASCs, naïve medium (without bacteria) or conditioned media (with bacteria) were recovered. Bacteria were incubated with these media again for 6 hours, but no significant difference in bacterial permeabilization was pointed out, suggesting that ASC-conditioned media were not sufficient to trigger such antibacterial effect (Figure 3(a)). Since the lack of difference could be due to the way the supernatants were processed, we performed a 0.4 *μ*m transwell assay to test indirect and direct actions of ASCs on bacteria ([Supplementary-material supplementary-material-1]). Comparing outer parts of transwell, we confirmed the direct action of ASCs on bacterial permeabilization of the three tested strains. Comparing inner parts of transwell, we demonstrated a significant increase in the proportion of PI-positive bacteria for *Fn* and *Sg*; however, this proportion was significantly lower than for direct action (Figure 3(b)). Six hours with different doses of antibiotics ampicillin or metronidazole, ASCs significantly decreased the number of *Fn* CFUs compared to controls (Figure 3(c)). Furthermore, our data also suggested an action of ASCs to potentiate ciprofloxacin on *Ec* ([Supplementary-material supplementary-material-1]). Together, these results demonstrate that contact is important for ASC antibacterial effects leading to bacteria growth reduction, membrane permeabilization, and sensitization to antibiotics.

### 3.5. ASC ROS Production after Coculture with Bacteria

**Figure 4:**
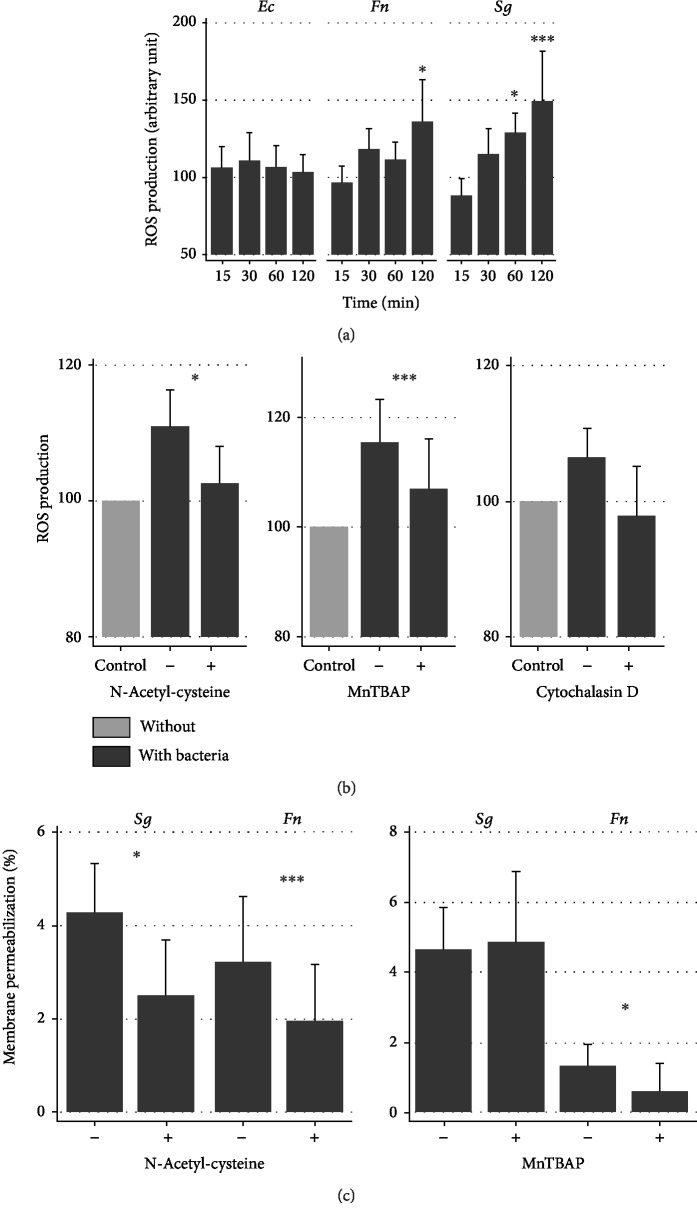
The increase in intracellular ROS is dependent on bacterial incubation duration and bacterial strain. (a) After ASC staining with the ROS probe H_2_DCFDA, cells were cultured with *Ec*, *Fn*, or *Sg* for 15, 30, 60, or 120 minutes and fluorescence was measured by flow cytometry (*N* = 6). (b) Before and during infection, the culture medium was supplemented with antioxidant (NAC, MnTBAP) or cytochalasin D, and the cells were incubated with or without *Sg* for 60 minutes and ROS production was measured using the H_2_DCFDA probe (*N* = 6). The mean fluorescence values were given (arbitrary units). (c) Bacterial wall permeability of *Sg* and *Fn* induced by ASCs with or without antioxidants (NAC, MnTBAP) normalized according to the respective medium without ASCs and with or without antioxidants (*N* = 8).

Reactive oxygen species (ROS) production is known to play a major role in antimicrobial host defense mechanisms. ROS generated by ASCs were then measured over time after contact with bacteria. While no difference was detected after 15 minutes, there was a significant increase in ROS production 30 and 60 minutes after contact with *Sg* and *Fn* (Figure 4(a)). For *Ec*, the ROS production was lower than the other strains (Figure 4(a)). As *Sg* infection provided the strongest ROS production, this strain was used for further analyses. The antioxidants NAC and MnTBAP significantly inhibited the *Sg* ROS production after 6 hours of incubation with ASCs. A tendency to reduce ROS was also observed using cytochalasin D, a potent inhibitor of actin polymerization, classically used to inhibit phagocytosis (Figure 4(b)). The use of antioxidants tended to decrease the bacterial membrane permeability of *Sg* and *Fn* even if no effect on bacterial CFUs was found. These results show that depending on bacteria strain, the ROS production by ASCs may participate in bacterial membrane permeabilization.

### 3.6. ASCs Displayed Bacterial Internalization and Phagocytic Activities

**Figure 5:**
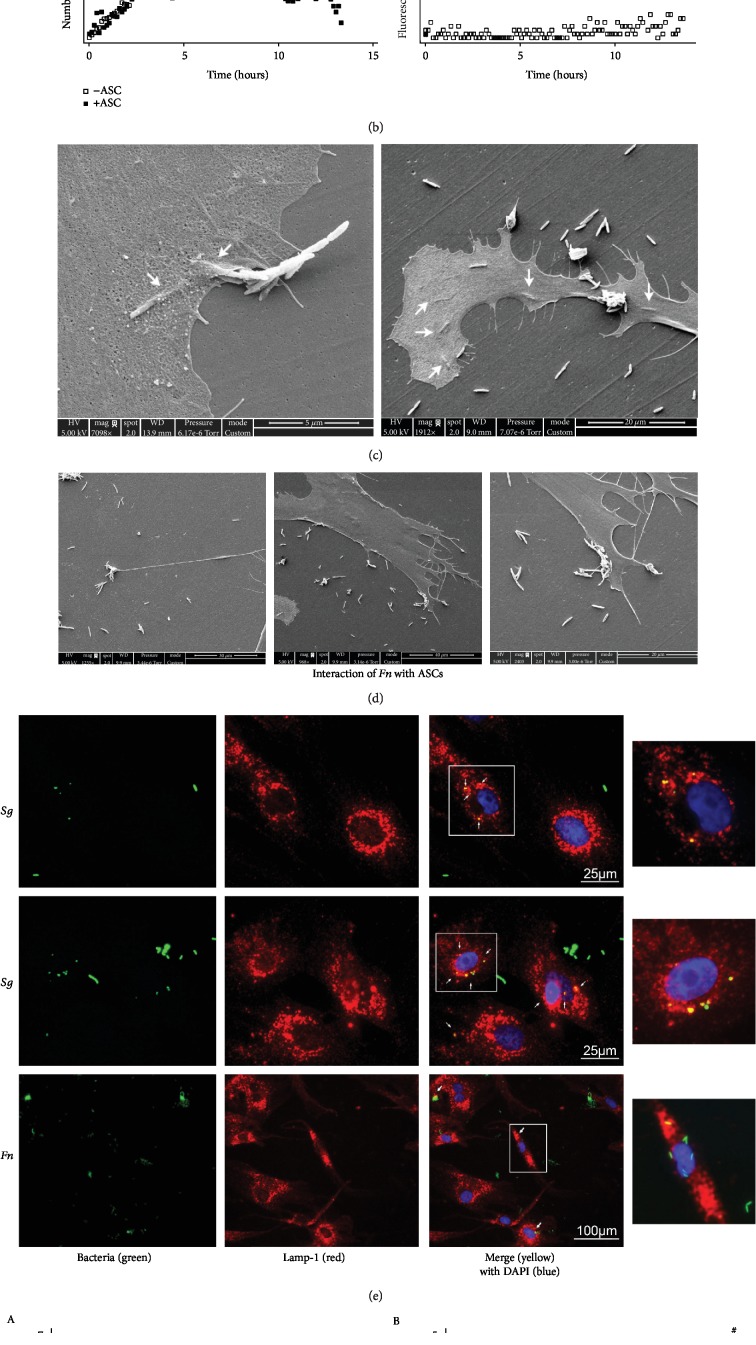
ASCs exhibited phagocyte-like activities. (a) The *Sg* bacteria were stained with CFSE (green) and cells with CellTrace Far Red (red) before time-lapse microscope captures at 0, 6, and 12 hours of incubation in contact. Interactions between bacteria and cells merge as yellow (*N* = 4). (b) The number of green particles was computerized using ImageJ over time (A), and the number of yellow pixels were counted (B). (c) Some SEM acquisitions showing *Fn* penetration in ASCs and *Fn* cell inclusion (white arrows). (d) *Fn* appears in SEM acquisitions as interacting with specific membrane areas. (e) Several bacterial strains or PBS (isotype) were stained with the green pH-sensitive marker (pHrodo). Cells were incubated at 1 : 100 infection ratio for 1 hour, with or without the cytoskeletal inhibitor, cytochalasin D, at 0.4 *μ*M. (e) ASCs were infected with CFSE-stained (green) bacteria. Cells were then fixed and immunostained with anti-LAMP-1 (red). The merge appears in yellow (white arrows to the right). Nuclei were stained with DAPI (blue) (*N* = 4). Magnifications (white frames) were provided on the right column. (f) Mean fluorescence and percentage of green positive cells were shown in A and B, respectively. Code “@” indicates a significant difference between the experimental conditions with cytochalasin D and the respective isotype control, and code “#” indicates a significant difference between the experimental conditions without cytochalasin D and the respective isotype control (*N* = 7).

Using ASC membrane staining and CFSE-labelled *Sg*, time-lapse acquisitions suggested ability of ASCs to capture and to internalize bacteria (Movies [Supplementary-material supplementary-material-1] and [Supplementary-material supplementary-material-1]). In the presence of ASCs, we observed a decrease in the number of bacteria from about 6 hours in comparison to bacteria alone (Figures 5(a) and 5(b), A). From about 15-30 minutes, bacterial interaction with the membrane remained constant over time (Figure 5(b), B). SEM acquisitions also suggested that *Fn* may be internalized by ASCs (Figure 5(c)) and that ASCs exhibited preferential attachment areas, including some cellular extensions (Figure 5(d)). Intracellular colocalization between bacteria and LAMP-1 (ubiquitously expressed in lysosomes and late endosomes and involved in lysosomal stability and integrity [16]) staining suggested that *Sg* and *Fn* were included inside phagolysosomes (Figure 5(e); [Supplementary-material supplementary-material-1]). When bacteria were labelled with a pH-sensitive dye (whose intensity of fluorescence increased dramatically when the pH decreased), we observed after incubation with *Sg* and *Fn* a significant increase of fluorescent-positive cells and mean fluorescence. This increase was almost abolished when the inhibitor of actin polymerization cytochalasin D was used (Figure 5(f)). Thus, ASCs can elicit phagocytosis events to further address bacteria to lysosomal degradation.

### 3.7. Relevance of the Broad-Spectrum Antibacterial Effect of ASCs on Periodontal Disease

**Figure 6:**
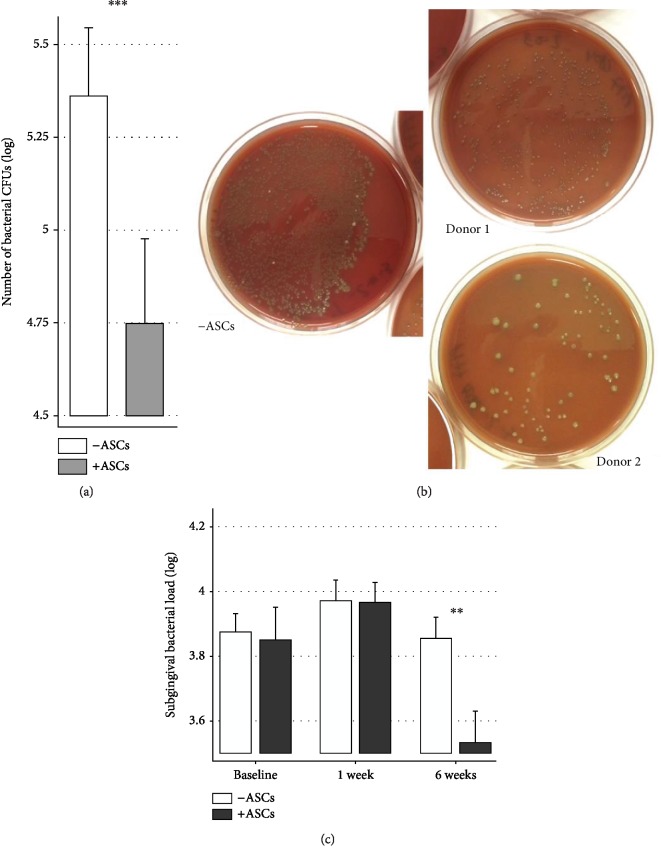
ASCs exhibited antibacterial effects against human and murine periopathogens. (a) Human bacterial subgingival samplings were incubated for 6 hours without (white bar) or with (grey bar) ASCs, and the number of bacterial CFUs was determined. Five ASC donors and 5 subgingival bacterial samples, for 12 unique combinations, were tested. ^∗∗∗^*p* < .001. (b) Representative results from incubation of ASCs with subgingival specimen from one patient. The control is on the left, example of two ASC donors incubated with the same subgingival sample on the right. (c) Quantification of subgingival anaerobic bacterial flora (expressed in CFU log) from a murine model of periodontitis after periodontal defects grafting with or without ASCs. ^∗∗^*p* < .01 (*N* = 6 by time point).

This antibacterial activity of ASCs was confirmed using human subgingival samples. After incubation with these “wild” periopathogenic samples, culture medium from ASCs significantly decreased the CFU formation (Figures 6(a) and 6(b)). In a mouse model of pathogen-induced periodontitis [15], the number of CFUs was significantly decreased (Figure 6(c)) on the ASC-grafted side compared to the control side (3.53 ± 0.37 versus 3.86 ± 0.24, *p* = .002). Two main bacterial species, gram-positive and catalase-negative, had been identified as *Staphylococcus xylosus* and *Streptococcus sciuri*. All together, these data suggest an ability of ASCs to reduce bacterial load in periodontal disease contexts.

## 4. Discussion

We have highlighted antibacterial effects of ASCs on several strains of bacteria. Our data suggest that multiple mechanisms are involved including bacterial membrane permeability, ROS production, and phagocytosis. This effect is positively correlated with the number of cells, negatively correlated with the number of initial bacteria, and maximum at 6 hours under the culture conditions used for these experiments.

Changes in bacterial permeability induce changes in the bacteria functions [17]. The depolarization of gram-positive thus mimics the phenomena observed during contact with some antibiotics [17]. Although surprising, the hyperpolarization observed for *Ec* is also a marker of the loss of bacterial viability as previously reported, for example, during alkaline stress, proton capture, and ATP hyperconsumption [18]. Increasing bacterial permeability may be a strategy to promote antibiotic action on resistant strains [19, 20]. Indeed, we demonstrated that ASCs enhanced the action of ampicillin and metronidazole on *Fn*. This is in favor of the presence of ASC-secreted molecules able to bind the bacterial outer lipid double layer thus disrupting the membrane organization [21]. This action could be highly relevant for tissue regeneration taking place in an environment where residual bacteria can be resistant or few sensitive to antibiotics (e.g., *Staphylococcus* spp. resistant to methicillin in skin ulcerations in diabetic patients). Many cationic peptides, such as defensins, have been shown to be potentially involved in this action [22]. Nevertheless, the increase in bacterial permeability does not necessarily lead to the loss of bacterial viability [23], a paradigm different to that observed in eukaryotic cells. Propidium iodide highlights bacterial permeability and is therefore a sensitive marker of cell damage, but it is not an indicator of cell death in stressed bacteria—although we showed that both events correlate [23]. The absence of modification of the parameter *λ* during the reculture phase of bacteria exposed to ASCs supports the fact that ASC effect is bactericidal rather than bacteriostatic.

ASCs can generate oxygenated free radicals, such as macrophages, via a NADPH-dependent mechanism [24]. The production of oxygenated free radicals may be involved in bacterial permeabilization. Their production could be related to bacterial internalization since the noninvasive strain *Ec* (ATCC25922) does not show an increase in the signal of H_2_DCFDA or pHrodo, compared to *Fn* and *Sg* strains. The use of cytoskeleton inhibitor cytochalasin D also decreased them. Hydrogen peroxide could be involved in this effect since strains expressing catalase only were less sensitive to ASC action ([Supplementary-material supplementary-material-1]). H_2_O_2_ is responsible for direct oxidative damages to many pathogens and acts as a substrate for many oxidative molecules [25]. As we suggested by multivariate analysis, an increase in body mass index increased the bactericidal effect of ASCs. Our results are in line with the increase in phagocytic activity reported for stromal vascular fraction of adipose tissue from obese compared to lean mice [26]. Moreover, ASCs from obese patients exhibit significantly higher levels of ROS compared to ASCs from a nonobese subject [27]. ROS activities are however pleiotropic. They can also act as a second messenger in intracellular signal transduction and interfere with cellular processes, including proliferation, migration, lineage commitment, and paracrine secretions [28]. They are also reported to enhance the regenerative potential of ASCs, by supporting angiogenesis through VEGF production [29] and to induce ASC differentiation into adipocytes [30].

Although phagocytosis may have lower impact than the indirect cell effects of ASCs, bacterial internalization may act as a trigger event. Kriebel et al. reported in an anaerobic model that *Fn* is able to invade bone marrow MSCs and to stimulate interleukin 8 secretion [31]. Properties of *Candida parapsilosis* ingestion and killing were previously demonstrated on 3T3-L1 preadipocyte cells using acridine orange and crystal violet as indicators of viability [32]. We showed in this study that both gram-negative and positive bacteria were internalized by ASCs into their phagolysosomes. ASCs share the expression of a great number of genes with macrophages [11]. SEM acquisitions suggested preferential cell membrane fixation sites for *Fn*. ASCs were shown to express pattern recognition receptors, the Toll-like receptor family (TLRs), involved in detecting bacterial components and activating immune cells [33]. ASCs express TLR-1 to TLR-6 and TLR-9 [34]. Lipopolysaccharides from *Ec* and peptidoglycans from *Sa* increase osteogenic differentiation of ASCs, and hypoxic culture conditions increased expression of TLR-1, 2, 5, and 9. TLR-1 recognizes a broad range of pathogens, TLR-2 gram-positive bacterial components as peptidoglycans, TLR-5 bacterial flagellin, and TLR-9 the CpG motif of bacterial DNA [34]. Taken together, the beneficial effects of ASCs could be modulated according to the partial oxygen pressure and the microbiome of the environment.

Data reporting antibacterial effects of ASCs are sparse [20, 35], and mechanisms of action may be inferred from those reported for other types of MSCs [35]. Literature reports that the antibacterial effects of MSCs may be linked to cell phagocytosis [32], antibacterial peptide LL-37 production [36, 37], hepcidin [35], *β*-defensin 2/TLR-4 [38], lipocalin-2 [39], and iNOS and IDO system involvement with tryptophan depletion in the environment [40]. The antibacterial effects of MSCs may be reinforced by the autoparacrine secretion of proinflammatory cytokines such as IL-17 [41] or IFN-*γ* [40]. Recent works identify the antimicrobial peptide of the cathelicidin family, LL-37, as responsible for the anti-*Sa* activity of adipocytes [37] and partly responsible for an antibacterial effect of ASCs, reinforced by inflammatory cytokines [20]. In addition to antimicrobial peptide production, our study reveals that ASCs act through multiple and combined actions depending on the context and bacterial strain. ASCs may alter bacterial membrane integrity leading to reduction of cell growth and viability, abnormality in cell division, and sensitization to antibiotics. ASCs may also use ROS production and phagocytosis to trigger their antibacterial effects. Optimized effects require contact between ASCs and bacteria.

MSCs are able to protect against sepsis by stimulating the activity of circulating monocytes, increasing bacterial clearance, and thus protecting against septic shock [8]. We showed here that ASCs induced the decrease in the number of bacterial colonies from the sulcus sample in an *in vivo* model of mouse periodontitis. However, interaction with the actors of the immunity system is undoubtedly a mechanism that remains to be explored [8].

## 5. Conclusions

Taken together, our results highlight for the first time a broad range of antibacterial action of ASCs by phagocytosis, secretion of oxygenated free radicals and antibacterial molecules. In an original and unique manner, this study stands out on a broad range of bacteria, with 4 gram-negative and 4 gram-positive strains. The increase in bacterial permeability resulting in an increase in antibiotic sensitivity is also highly relevant for environment where residual bacteria can be resistant or few sensitive to antibiotics. Given the development of cell therapy, particularly in applications in which the presence or persistence of microbial components could impact the outcome of the procedure, these data are in line with the development of new therapeutic strategies based on ASC transplantation, appropriated in an immune-dysbiotic tissue context such as periodontitis. The comparison of the antibacterial properties of ASCs with other cell types (i.e., other sources of MSCs, fibroblasts), as well as the impact of the native tissue microenvironment on ASC antibacterial effects, could be the subject of additional investigations.

## Figures and Tables

**Table 1:** ASCs modified growth rate of bacteria. Parameters were estimated using bacterial growth modeling with the modified Gompertz equation for three bacterial strains (*Ec*, *Sg*, and *Sa*). Bacterial growth was measured by monitoring optical density at 600 nm for 12 hours. DO_600_ = Aexp(−exp(*μe*/*A*(*λ* − t) + 1)). Parameters: *t* was the time, *μ* the growth rate, *A* the maximum optical density, and *λ* the lag time. We observed no significant difference in *A* and *λ* parameters.

Strain	Group	*N*	*μ* Mean ± SD	*p* value
*Ec*	Without ASCs	7	0.82 ± 0.13	.03
	With ASCs		0.74 ± 0.14	

*Sa*	Without ASCs	6	0.63 ± 0.25	.04
	With ASCs		0.58 ± 0.23	

*Sg*	Without ASCs	6	0.59 ± 0.24	.04
	With ASCs		0.45 ± 0.17	

## Data Availability

The data used to support the findings of this study are included within the article and within the supplementary information files.

## Supplementary Materials

Supplementary 1 Table S1: bacterial strains. Table S2: characteristics of adipose tissue donors. Table S3: ASCs modified bacteria size and granularity. Table S4: membrane changes induced by ASCs do not permit the passage of *β*-galactosidase. Figure S1: transwell assay. Figure S2: ASCs exhibited an antibacterial activity, maximal at 6 hours. Figure S3: ASCs exhibited antibacterial activity dependent on the number of initial bacterial load. Figure S4: ASCs induced a shift in bacterial PI fluorescence. Figure S5: broad-spectrum antibacterial effect of ASCs was dependent on initial bacterial load. Figure S6: ASCs modified size and granularity of bacteria. Figure S7: ASCs did not modify the bacterial surface. Figure S8: ASCs to potentiate ciprofloxacin on *Ec*. Figure S9: *Sg* and *Fn* inside the phagolysosomes.

Supplementary 2 Movie S1: time-lapse confocal movie for 14 hours, without (left) and with (right) ASCs.

Supplementary 3 Movie S2: example of ASC pseudopod formation (white arrow).
