# Supplementary material for: Broad-Spectrum Antibacterial Effects of Human Adipose-Derived Stromal Cells
Source: Stem Cells Int. 2019 Oct 29;2019:5389629. doi: 10.1155/2019/5389629 (PMC6855043; doi:10.1155/2019/5389629)
Supplement: Supplementary 1 — Table S1: bacterial strains. Table S2: characteristics of adipose tissue donors. Table S3: ASCs modified bacteria size and granularity. Table S4: membrane changes induced by ASCs do not permit the passage of β-galactosidase. Figure S1: transwell assay. Figure S2: ASCs exhibited an antibacterial activity, maximal at 6 hours. Figure S3: ASCs exhibited antibacterial activity dependent on the number of initial bacterial load. Figure S4: ASCs induced a shift in bacterial PI fluorescence. Figure S5: broad-spectrum antibacterial effect of ASCs was dependent on initial bacterial load. Figure S6: ASCs modified size and granularity of bacteria. Figure S7: ASCs did not modify the bacterial surface. Figure S8: ASCs to potentiate ciprofloxacin on Ec. Figure S9: Sg and Fn inside the phagolysosomes. [file 5389629.f1.pdf]

Supplementary Information for

Broad spectrum antibacterial effects of human adipose-derived stromal cells

Paul Monsarrat, Philippe Kémoun, Louis Casteilla, Valérie Planat-Bénard

Correspondence to: paul.monsarrat@univ-tlse3.fr

Supplementary Tables **2**

Table S1: Bacterial strains 2

Table S2: Characteristics of adipose tissue donors 2

Table S3: ASCs modified bacteria size and granularity. 3

Table S4: Membrane changes induced by ASCs do not permit the passage of β-galactosidase. 3

Supplementary Figures **4**

Figure S1: Transwell assay. 4

Figure S2: ASCs exhibited an antibacterial activity, maximal at 6 hours. 4

Figure S3: ASCs exhibited antibacterial activity dependent on the number of initial bacteria load. 5

Figure S4: ASCs induced a shift in bacterial PI fluorescence. 5

Figure S5: Broad spectrum antibacterial effect of ASCs was dependent on initial bacterial load. 6

Figure S6: ASCs modified size and granularity of bacteria. 7

Figure S7: ASCs did not modify the bacterial surface. 7

Figure S8: ASCs to potentiate ciprofloxacin on *Ec*. 8

Figure S9: Sg and Fn inside the phagolysosomes. 8

Supplemental Movies **9**

Movie S1: Timelapse confocal movie during 14 hours, without (left) and with ASCs (right). 9

Movie S2: Example of ASCs pseudopods formation (white arrow). 9

Supplementary Tables

Table S1: Bacterial strains

This table presents the bacterial strains used in this study, their origin (CIP or ATCC collection). The peroxidase, catalase, superoxide dismutase protein expression is provided according to UniprotKB data (http://www.uniprot.org/uniprot/) according to the classification of the Committee of The International Union of Biochemistry and Molecular Biology (NC-IUBMB). The conditions of culture, type of agar and atmosphere, are given. Incubations were performed at 37°C. For anaerobic culture, the strains were placed in jars (GENbox anaer, Biomérieux).

Bacterial strain	Reference	PEROX	CAT	SOD	Culture condition
Lactobacillus casei	CIP 103137	No	No	No	BHI Agar plates - Aerobic
Streptococcus sanguinis	CIP 55128	Yes	No	Yes	TCS Agar plates with 10% defibrinated sheep blood, 5µg/ml hemin and 1µg/ml menadione - Aerobic
Staphylococcus aureus	ATCC 888807	Yes	Yes	Yes	BHI Agar plates - Aerobic
Escherichia coli	ATCC 25922	Yes	Yes	Yes	BHI Agar plates - Aerobic
Enterococcus faecalis	ATCC 29212	Yes	Yes	Yes	BHI Agar plates - Anaerobic
Porphyromonas gingivalis	ATCC 33277	Yes	No	Yes	TCS Agar plates with 10% defibrinated sheep blood, 5µg/ml hemin and 1µg/ml menadione - Anaerobic
Fusobacterium nucleatum	ATCC 25586	No	No	No	
Prevotella intermedia	CIP 103607	Yes	No	No	

Abbreviations: BHI: Brain Heart Infusion; CAT: catalase; PEROX: peroxidase; SOD: superoxide dismutase; TCS: trypticase soy.

Table S2: Characteristics of adipose tissue donors

	BMI < 25	25 ≤ BMI < 30	BMI ≥ 30	All
**Number of subjects**	18	19	5	42
**Age (years)**	40.7 ± 8.4	41.4 ± 9.4	40.9 ± 19.6	41.1 ± 10.3
**Gender (M/F)**	1/17	1/18	0/5	2/40
**Weight (kg)**	66.8 ± 6.6	71.6 ± 6.8	80 ± 8.5	70.5 ± 8.0
**Body Mass Index (kg/m²)**	23.4 ± 1.2	26.9 ± 1.5	31.4 ± 0.9	26.0 ± 2.9

Abbreviation: BMI: Body Mass Index. Statistics are expressed as mean ± standard deviation.

Table S3: ASCs modified bacteria size and granularity. This table shows FSC and SSC values for every strain (with or without ASC incubation). These values were standardized compared to obtained values from added microbeads.

	FSC			SSC		
**Strain**	Without ASC	With ASC	**Sig**	Without ASC	With ASC	**Sig**
Ec	43.8 ± 6.55	44.2 ± 5.82	ns	40 ± 3.72	39.9 ± 5.57	ns
Ef	22.7 ± 6.95	27.8 ± 6.22	**	34.4 ± 7.65	35.9 ± 5.81	ns
Fn	27.4 ± 3.78	32.3 ± 3.96	***	39.3 ± 4.45	41.7 ± 6.68	***
Lc	40.5 ± 4.64	41.1 ± 3.88	ns	50.4 ± 1.39	49.4 ± 4.34	ns
Pg	27.2 ± 5.54	25.2 ± 4.26	ns	36 ± 4.79	34.1 ± 3.68	*
Pi	27.4 ± 3.93	28.5 ± 3.77	***	33.5 ± 2	33.5 ± 2.38	ns
Sa	84.5 ± 6.41	79.3 ± 5.32	***	64.8 ± 3.66	62.7 ± 3.85	***
Sg	25.4 ± 6.38	29.6 ± 6.16	***	35.2 ± 3.58	38.2 ± 6.91	***

Abbreviations: FSC: Forward Scatter; ns: not significant; SSC: Side Scatter. For strain abbreviations please report to Table S1, above.

Table S4: Membrane changes induced by ASCs do not permit the passage of β-galactosidase. After incubation of the bacteria for 6 hours, with or without contact with the ASCs, the supernatants were recovered after double centrifugation to remove remaining bacteria. After contact with the ONPG substrate for β-galactosidase, the reaction was carried out, monitored by spectrophotometry to calculate the enzymatic activity in variation of optical density per unit of volume per minute. To generate positive control, a solution of *Ec* was lysed using Tween 20 and chloroform.

**Strain**		**N**	**Activity**	**Sig**
Ec	Without ASCs	8	6.5 ± 1.9	*
	With ASCs		3.3 ± 3.2	
Positive control	Without ASCs	8	6.0 ± 2.7	***
	With ASCs		22.3 ± 2.0	

Supplementary Figures

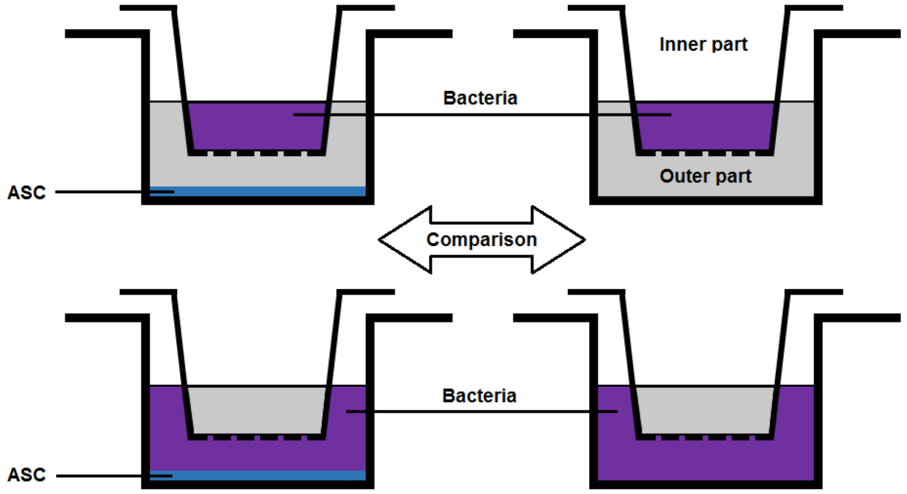

Figure S1: Transwell assay. Transwell inserts were used with 0.4μm PET membranes. To test the indirect action of ASCs, we compared bacteria from the inner parts of inserts with cells versus without cells (upper line). To test the direct action of ASCs, we compared bacteria from the outer parts of inserts with cells versus without cells (bottom line).

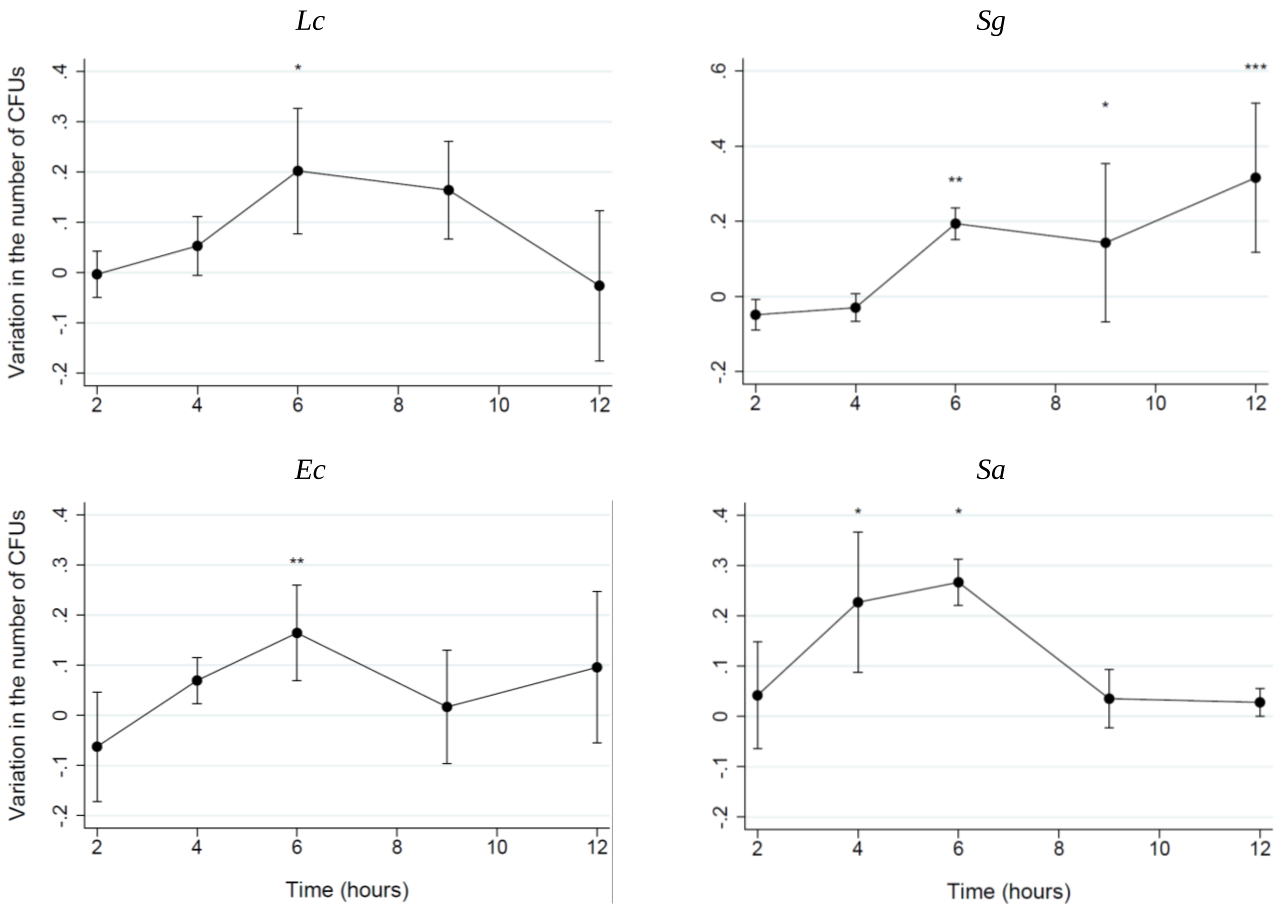

Figure S2: ASCs exhibited an antibacterial activity, maximal at 6 hours. The difference between the log number of bacterial colony forming units (CFUs) without contact and with contact with ASCs was measured at different timepoints.

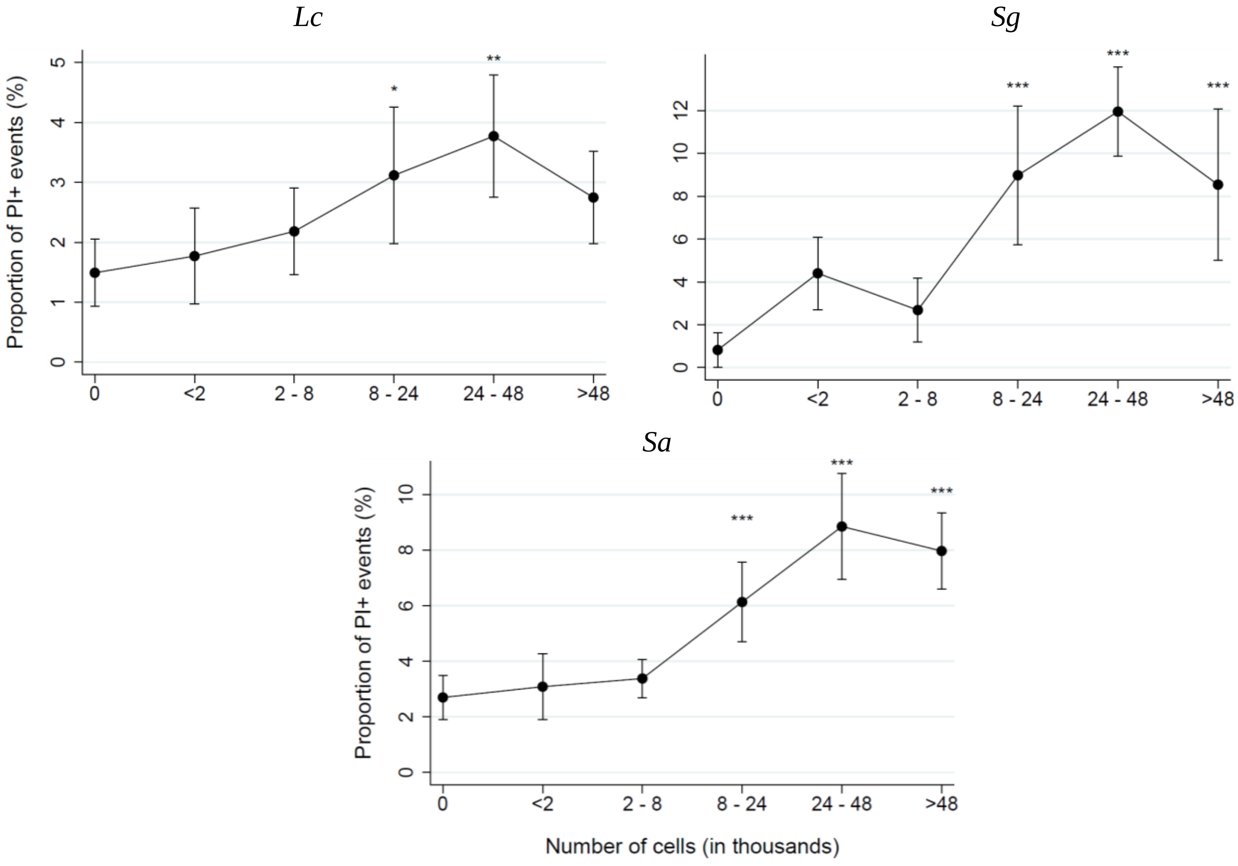

Figure S3: ASCs exhibited antibacterial activity dependent on the number of initial bacteria load. Different number of cells in a 12-wells plates were incubated with the same number of bacteria during 6 hours. Bacteria were stained Syto-62® and propidium iodide (PI), then analyzed by flow cytometry to obtain the proportion of PI positive bacteria (membrane permeability (%)).

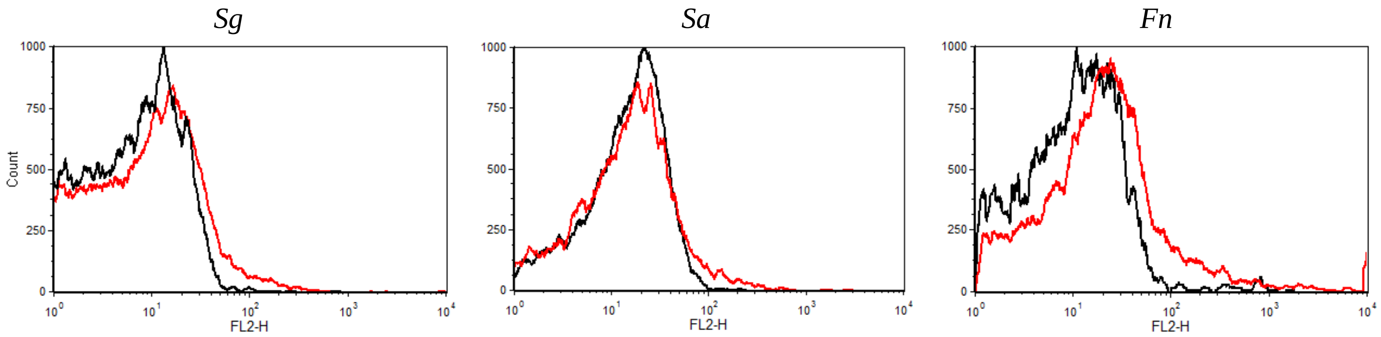

Figure S4: ASCs induced a shift in bacterial PI fluorescence. Fluorescence histogram was plotted for bacteria alone (black curve) or in contact with ASCs (red curve).

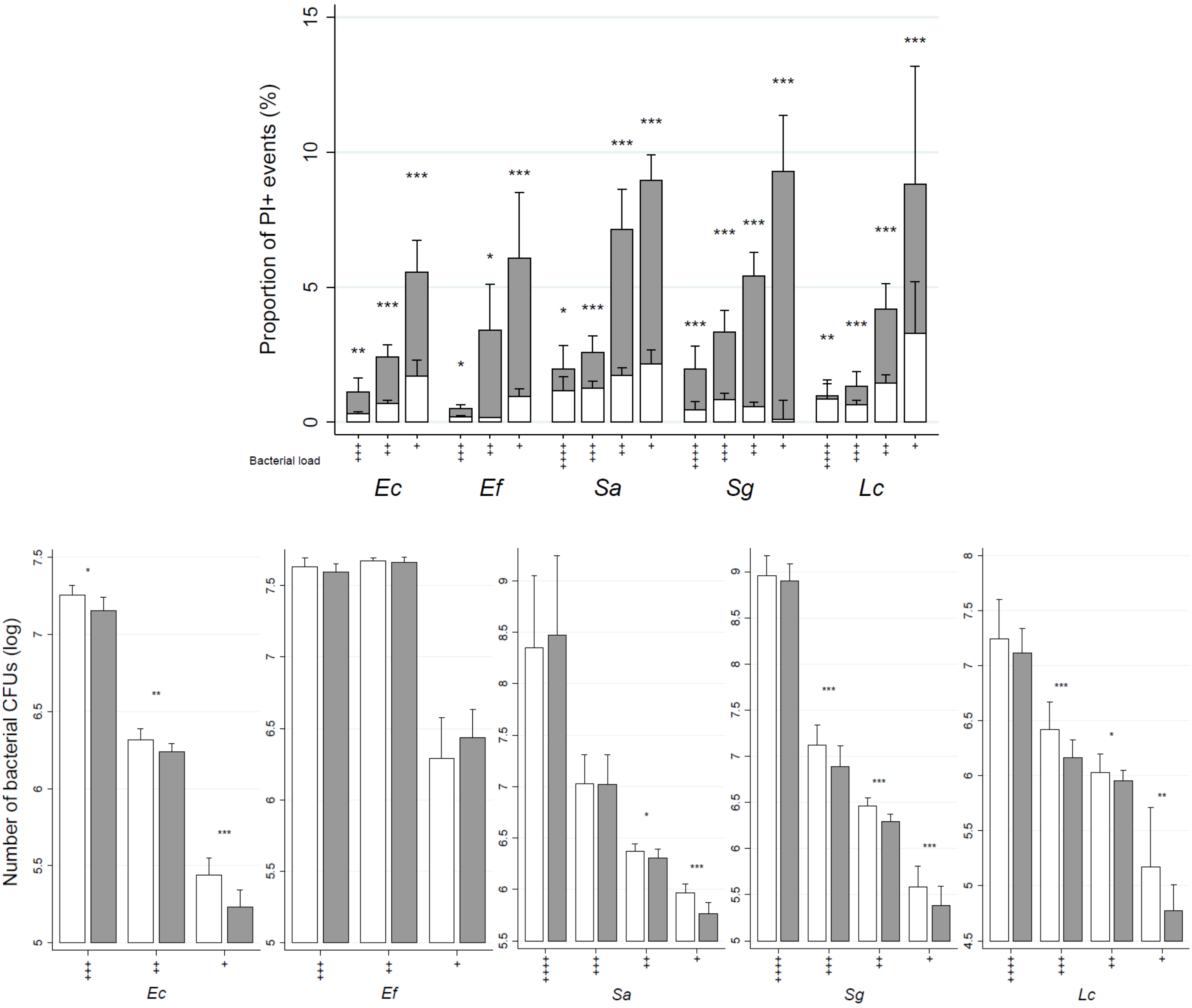

Figure S5: Broad spectrum antibacterial effect of ASCs was dependent on initial bacterial load. Culture medium was incubated with various initial dose of bacteria, with ASCs (grey bar) or without (white). **A -** Bacterial membrane permeability was assessed by flow cytometry as previously described (membrane permeability (%). **B -** Bacterial CFUs were counted as already detailed.

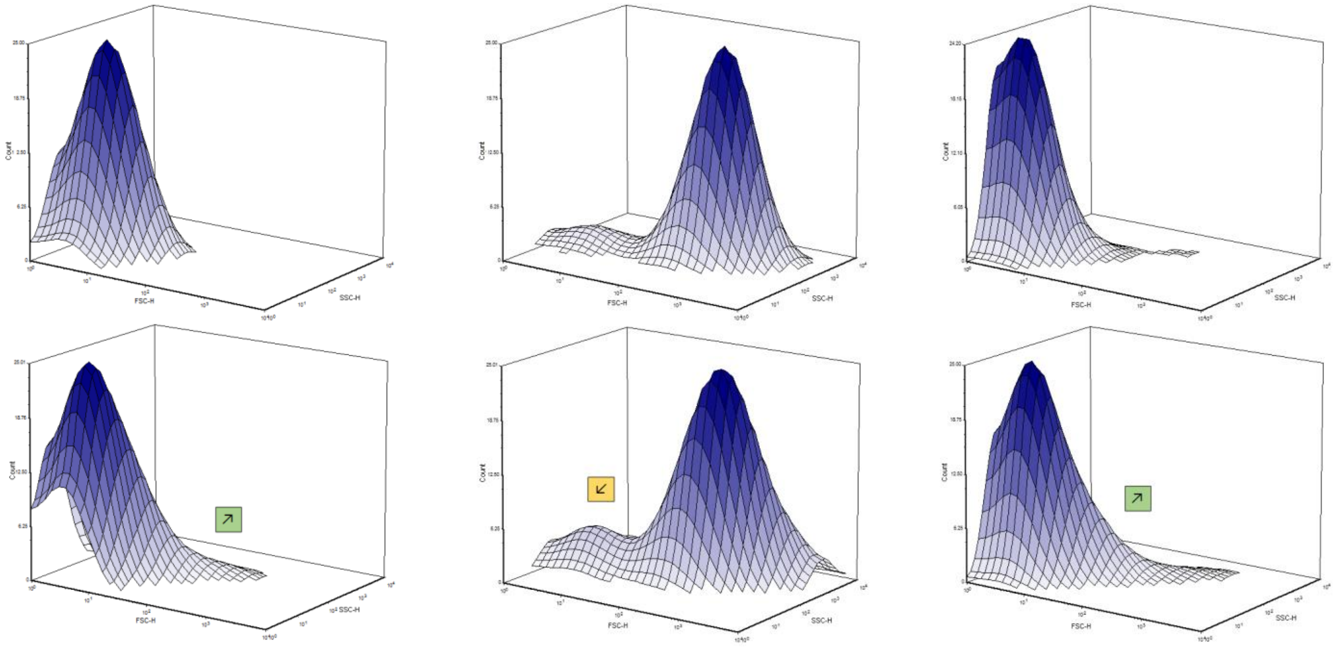

Sg *Sa* *Fn*

Figure S6: ASCs modified size and granularity of bacteria. These multidimensional histograms show the variation of SSC and FSC for three bacterial strains (*Sg*, *Sa* et *Fn*) without contact (upper) or without contact (below) with ASCs.

Sa

Fn

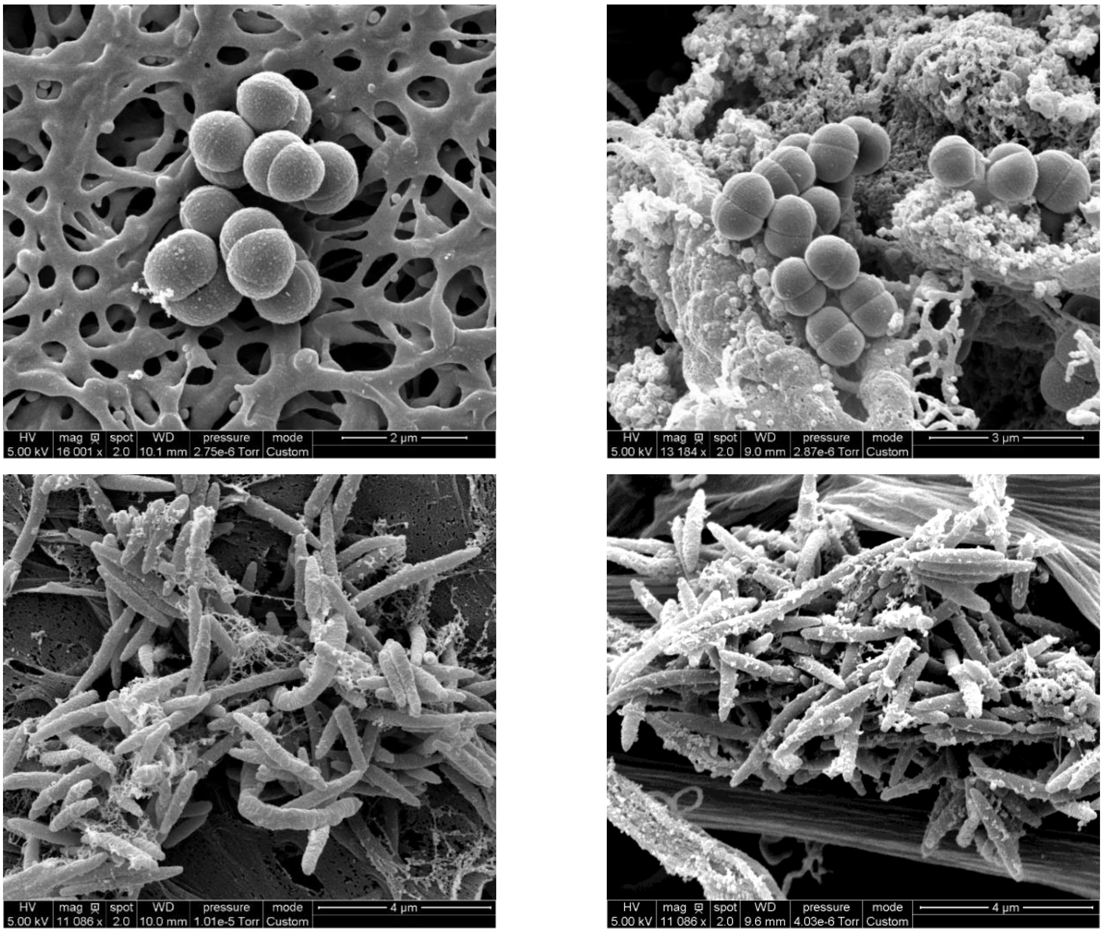

Without ASC **With ASC**

Figure S7: ASCs did not modify the bacterial surface. In SEM experiment, we observed no apparent changes in surface of bacteria (such as visible holes or blebs).

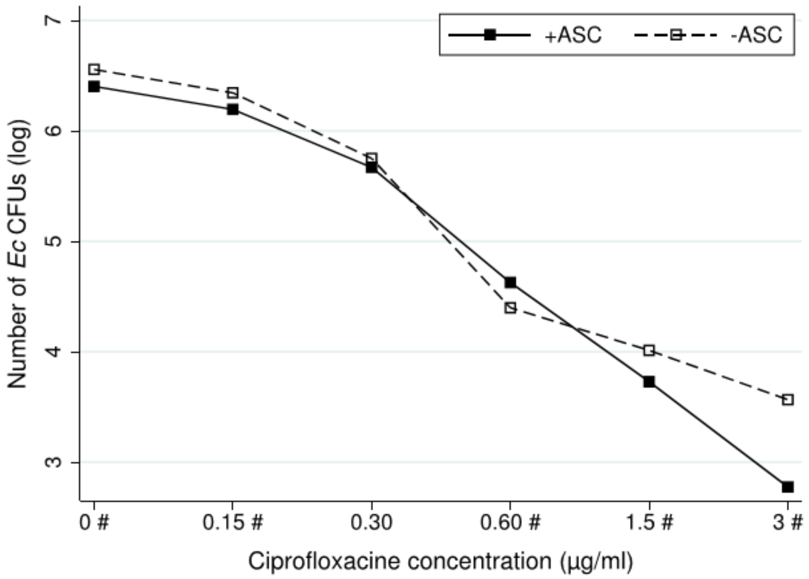

Figure S8: ASCs to potentiate ciprofloxacin on *Ec*. Symbol # indicates a significant difference between both groups.

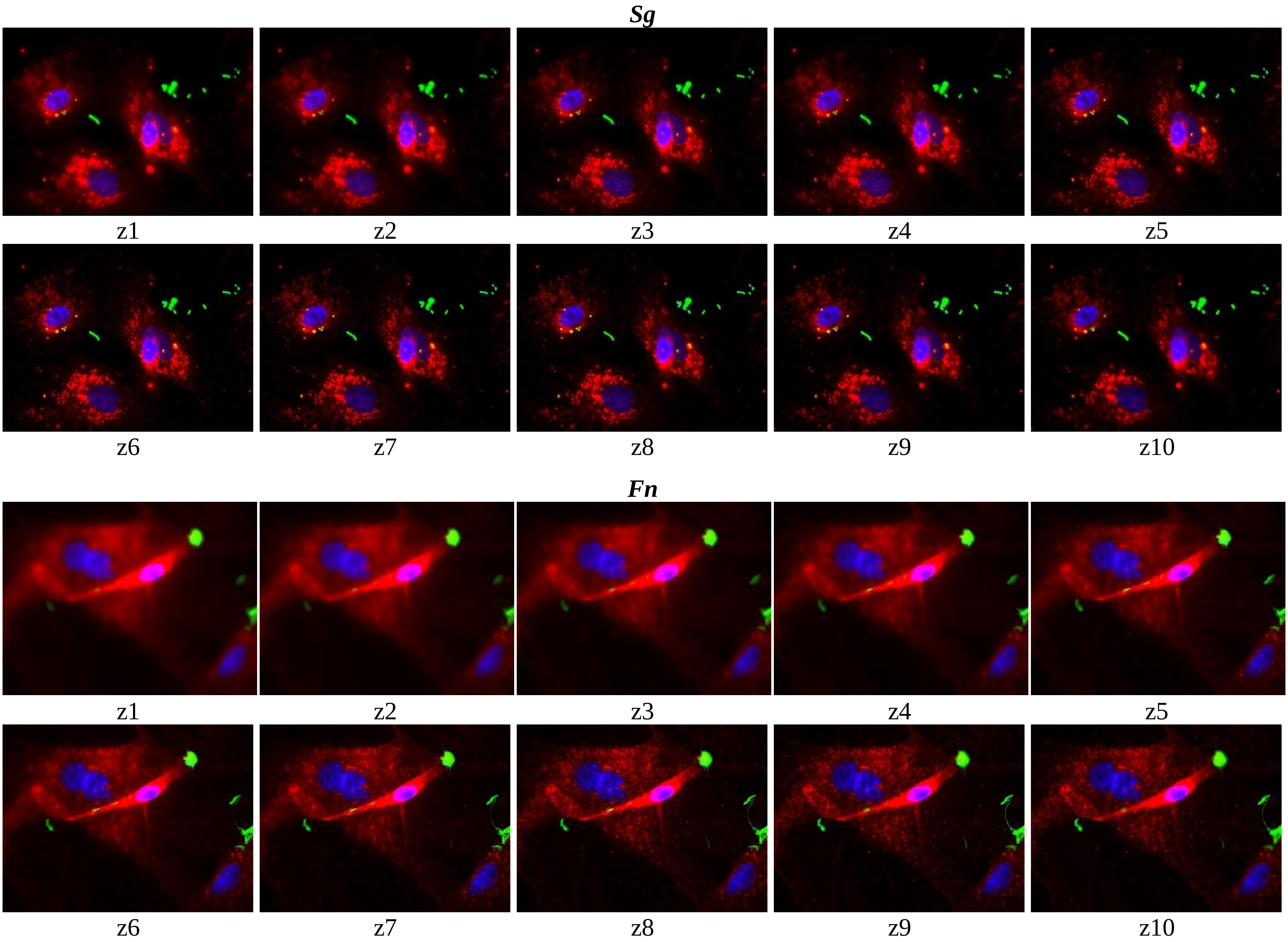

Figure S9: *Sg* and *Fn* inside the phagolysosomes. ASCs were infected with bacteria marked CFSE (green) at 1:100 for 1 hour. The cells were fixed and marked with LAMP-1 (red). The collocation appears in yellow. Z-stack acquisitions.

Supplemental Movies

Movie S1: Timelapse confocal movie during 14 hours, without (left) and with ASCs (right). The *Sg* bacteria were first marked with CFSE (green) and the cells with CellTrace far red (red). Interactions between bacteria and cells appear yellow.

Movie S2: Example of ASCs pseudopods formation (white arrow). The *Sg* bacteria were first marked with CFSE (green) and the cells with CellTrace far red (red). Interactions between bacteria and cells appear yellow.
